# Reconstruction of Post-Burn Neck Contractures: A Systematic Review and Meta-Analysis Comparing Surgical Techniques and Outcomes

**DOI:** 10.3390/jcm15145583

**Published:** 2026-07-16

**Authors:** Marko Jović, Milana Marinković, Jelena Rakocevic, Zoran Bukumirić, Milana Jurišić, Milan Jovanović, Milan Stojičić, Ljiljana Jovanović, Zoran Tabaković, David Savic, Jelena Jeremić

**Affiliations:** 1 Clinic for Burns, Plastic and Reconstructive Surgery, University Clinical Center of Serbia, 11000 Belgrade, Serbia; 2 Faculty of Medicine, University of Belgrade, 11000 Belgrade, Serbia; 3 Institute of Histology and Embryology “Aleksandar Đ. Kostić”, 11000 Belgrade, Serbia; 4 Institute for Medical Statistics and Informatics, 11000 Belgrade, Serbia; 5 Department of Clinical Research, Faculty of Health Sciences, University of Southern Denmark, 5230 Odenese, Denmark; 6 Institute for Cardiovascular Diseases “Dedinje”, 11000 Belgrade, Serbia

**Keywords:** burns, neck contracture, reconstruction, flaps, skin grafting, dermal substitutes

## Abstract

**Background:** Post-burn neck contractures continue to represent a complex reconstructive problem, with significant functional limitations and a marked impact on patients’ appearance and quality of life. This systematic review and meta-analysis aimed to assess surgical outcomes in adult patients undergoing reconstruction for post-burn neck contractures. **Methods:** A comprehensive search of Medline, Web of Science, and Scopus was performed up to January 2025. Studies reporting surgical management of post-burn neck contractures were included. Primary outcomes were functional and aesthetic improvement, while secondary outcomes included complications, recontracture, and disfiguring scarring. **Results:** In total, 46 studies comprising 1636 patients were included. Flap-based reconstruction was the most frequently applied approach. Overall, functional improvement was achieved in 92% of cases (95% CI: 89–94%), while satisfactory aesthetic outcomes were reported in 90% (95% CI: 85–93%). The pooled complication rate was 15% (95% CI: 11–20%), with higher rates observed following free flap reconstruction. The incidence of recontracture and disfiguring scarring remained relatively low, about 6%. Although functional outcomes were comparable across flap types, regional flaps appeared to offer the most favorable balance between aesthetic results and complication rates. In contrast, skin grafts and dermal substitutes showed less consistent outcomes and a higher tendency toward recurrence. **Conclusions:** Flap-based reconstruction remains the cornerstone of treatment for post-burn neck contractures. Surgical planning should be individualized, taking into account defect severity, tissue availability, and surgical expertise. Further prospective studies using standardized outcome measures are needed to strengthen current evidence.

## 1. Introduction

Burn injuries involving the head and neck region remain among the most challenging conditions in reconstructive surgery because of their profound functional and aesthetic consequences [1,2,3]. Following the acute phase of deep cervical burns, scar maturation frequently leads to contracture formation, particularly in patients with extensive full-thickness injuries [4,5]. Post-burn neck contractures may result in restricted cervical extension, mandibular and laryngeal deformities, difficult airway management, and impaired oral competence, substantially affecting daily functioning and quality of life [4,6,7,8,9,10,11]. In addition to functional limitations, cervical contractures are frequently associated with considerable psychosocial distress due to visible facial and neck disfigurement [6,7,8,9].

Beyond the immediate functional consequences, post-burn neck contractures represent an important cause of long-term disability among burn survivors [6,7,8,9,10]. The neck occupies a unique anatomical and functional position, serving as a dynamic interface between the face, airway, and upper torso. Consequently, cervical contractures may compromise not only the range of motion but also visual orientation, feeding, communication, airway access, and social interaction. The resulting impairment often extends far beyond physical limitations and may persist for decades after the initial injury.

Increasing attention has therefore been directed toward patient-centered outcomes following burn reconstruction. Previous studies have demonstrated that visible head and neck burn sequelae are associated with reduced satisfaction with appearance, social avoidance, impaired self-esteem, and long-term psychosocial distress [6,7]. Restoration of cervical mobility remains a primary surgical objective; however, contemporary reconstructive surgery increasingly aims to achieve broader goals, including recovery of cervical contour, improvement of facial harmony, facilitation of social reintegration, and enhancement of health-related quality of life. These considerations are particularly relevant in younger patients, who constitute a substantial proportion of individuals undergoing reconstruction for post-burn cervical deformities.

Despite advances in burn care and reconstructive surgery, management of post-burn neck contractures remains complex [5,8,12,13,14,15]. Successful reconstruction requires restoration of cervical mobility and contour while preserving vital structures and achieving satisfactory aesthetic integration with the surrounding tissue [5,11,12,14,16]. Reconstruction is further complicated by scarred adjacent tissues, limited donor-site availability in extensively burned patients, and the need to minimize secondary contracture and donor-site morbidity.

Current treatment strategies include both conservative and surgical approaches [17,18]. Conservative management may involve physical therapy, splinting, and compression therapy, whereas surgical treatment is based on scar release followed by reconstruction using skin grafts, dermal substitutes, local flaps, regional flaps, or distant free flaps [5,8,16,19,20,21,22]. More recently, regenerative and laser-based therapies have been explored as adjunctive modalities; however, the available evidence remains limited [23].

Recent advances in wound healing research have highlighted the growing potential of bioactive and immunomodulatory biomaterials to regulate inflammation, promote tissue regeneration, and improve the repair of complex wounds. Although these emerging approaches have demonstrated promising results in experimental and chronic wound settings, their application to mature post-burn scar contractures remains limited, where definitive treatment continues to rely predominantly on surgical reconstruction using established reconstructive techniques [24,25].

Although numerous reconstructive techniques have been described over the past decades, evidence-based guidance regarding the optimal reconstructive strategy remains limited. Most available studies consist of single-center retrospective case series involving relatively small patient cohorts, heterogeneous reconstructive techniques, and non-standardized outcome measures. Consequently, surgeons are often required to base reconstructive decisions on institutional experience rather than high-level comparative evidence. This heterogeneity in patient selection, treatment strategies, follow-up duration, and outcome assessment makes direct comparison between reconstructive modalities particularly challenging. To date, no comprehensive systematic review and meta-analysis has synthesized the available evidence regarding surgical outcomes after reconstruction of post-burn neck contractures in adults.

Therefore, this systematic review and meta-analysis aimed to synthesize the available evidence regarding surgical reconstruction of post-burn neck contractures in adults and to compare functional, aesthetic, and safety outcomes across different reconstructive strategies, with particular emphasis on identifying approaches that provide durable restoration of function and appearance. Unlike previous reports focusing on wound healing biology, regenerative approaches, or individual reconstructive techniques, the present study provides a quantitative comparison of different reconstructive modalities with pooled analyses of functional, aesthetic, and safety outcomes. By integrating the currently available evidence, this review offers a clinically oriented framework that may support reconstructive decision-making in patients with post-burn neck contractures.

## 2. Materials and Methods

This systematic review and meta-analysis were conducted in accordance with the PRISMA 2020 (Preferred Reporting Items for Systematic Reviews and Meta-Analyses) guidelines [26]. The study protocol was prospectively registered in the International Prospective Register of Systematic Reviews (PROSPERO; registration number CRD420251002768).

### 2.1. Outcomes

The primary outcome of this review was functional recovery following reconstruction of post-burn neck contractures. Secondary outcomes included aesthetic outcome, postoperative complications, recontracture, disfiguring scarring, and reintervention.

Because no standardized outcome assessment system currently exists for post-burn neck contracture reconstruction, both subjective and objective measures reported in the literature were considered eligible for analysis. Subjective assessment methods included surgeon- or patient-reported evaluations describing outcomes as satisfactory, good, excellent, or improved. Objective functional assessment included measurements of range of motion (ROM), atlanto-occipital extension angle, cervicomental angle, extent of cervical extension, and flap width after reconstruction [27,28,29].

The decision to pool both objective and subjective outcome measures was made a priori because no standardized or universally accepted outcome assessment system currently exists for post-burn neck contracture reconstruction. Restricting the analysis to a single assessment method would have excluded a substantial proportion of the available literature and reduced the representativeness of the evidence. Therefore, all clinically relevant outcome measures reported by the primary studies were considered eligible, while the method of outcome assessment (objective versus subjective) was systematically recorded and reported for each included study.

Postoperative complications were assessed at both donor and recipient sites, including complications related to tissue expansion when applicable. Both early postoperative complications and complications occurring during follow-up were included. Recontracture and disfiguring scarring were evaluated as separate outcome categories. Disfiguring scars were defined as hypertrophic, keloid, or aesthetically unacceptable scars reported by the authors. Reintervention was analyzed as a categorical outcome whenever data were available.

### 2.2. Search Strategy and Eligibility Criteria

A systematic literature search of Medline (via PubMed), Web of Science, and Scopus (Elsevier) was performed from database inception to 21 January 2025. The following search strategy was used: ((neck OR cervical) AND (contracture OR contractural OR contractured)) AND (burn OR burns) AND (treatment OR management OR intervention OR procedure). To maximize search sensitivity, reference lists of eligible studies were manually screened, while gray literature sources and Google Scholar were additionally searched.

Studies evaluating surgical treatment of post-burn neck contractures in adults (defined as patients aged ≥18 years) were considered eligible. Reconstructive modalities included direct closure, split-thickness or full-thickness skin grafts, dermal substitutes, local flaps, regional flaps, distant free flaps, laser therapy, and regenerative approaches. Randomized controlled trials, cohort studies, observational studies, clinical trials, and case series including at least 10 patients were eligible for inclusion. Studies involving mixed adult and pediatric populations were eligible only if the majority of participants (more than 50%) were adults (≥18 years) and separate neck-specific outcomes were reported.

Studies were excluded if they involved non-burn cervical contractures, failed to provide clearly identifiable neck-specific outcomes, or lacked an adequate description of treatment and outcomes of interest. Case reports, narrative reviews, systematic reviews, meta-analyses, animal studies, anatomical studies, and studies published in languages other than English were excluded.

### 2.3. Study Selection and Data Extraction

Two independent reviewers (M.M. and M.J.) independently screened titles and abstracts. Because eligibility could not be reliably determined from titles and abstracts alone, all potentially relevant records underwent full-text assessment according to the predefined inclusion and exclusion criteria. Disagreements regarding study inclusion were resolved through discussion with the senior reviewer (M.J.).

The following data were extracted from included studies: study characteristics, patient demographics, reconstructive technique, use of tissue expansion, number of reconstructive stages, follow-up duration, outcome assessment methods, functional and aesthetic outcomes, complications, recontracture, disfiguring scarring, and reintervention rates. Contracture severity was extracted whenever reported. However, because the included studies used heterogeneous definitions and non-standardized severity classifications, or did not report this variable at all, it could not be synthesized or incorporated into study-level comparisons.

### 2.4. Risk of Bias Assessment

Given that most included studies were retrospective case series or observational studies without control groups, methodological quality was assessed using the Joanna Briggs Institute (JBI) Critical Appraisal Checklist for Case Series, and JBI Check list for cohort studies. Two reviewers independently evaluated study quality, and disagreements were resolved by consensus with the senior reviewer. Studies were categorized as having low, moderate, or high risk of bias according to overall methodological quality and completeness of outcome reporting. Inter-reviewer agreement was substantial (Cohen’s κ = 0.82).

The certainty of evidence for each outcome was assessed using the GRADE (Grading of Recommendations Assessment, Development and Evaluation) approach. The certainty of evidence was evaluated across the domains of risk of bias, inconsistency, indirectness, imprecision, and publication bias.

### 2.5. Statistical Analysis

Statistical analysis was performed using the “meta”, “metafor”, and “dmetar” packages within the R statistical environment version 4.5.2 (R Foundation for Statistical Computing, Vienna, Austria). Pooled proportions were calculated using inverse variance methods with logit transformation under a random-effects model because of the anticipated clinical and methodological heterogeneity among studies.

Confidence intervals for individual studies were estimated using the Clopper-Pearson method. Statistical heterogeneity was assessed using Cochran’s Q test and quantified with the I^2^ statistic. Potential sources of heterogeneity were additionally explored using Baujat plots and influence analysis. Publication bias was evaluated using contour-enhanced funnel plots, trim-and-fill analysis, and Egger’s regression test (when at least 10 studies were available for a given outcome).

Because no standardized outcome assessment system exists for post-burn neck contracture reconstruction, pooled analyses were performed using clinically comparable endpoints rather than identical measurement instruments.

Sensitivity analyses were performed by excluding studies identified as influential during influence analysis to assess the robustness of pooled estimates. Statistical significance was defined as *p* < 0.05.

## 3. Results

### 3.1. Study Search and Study Characteristics

**Figure 1:**
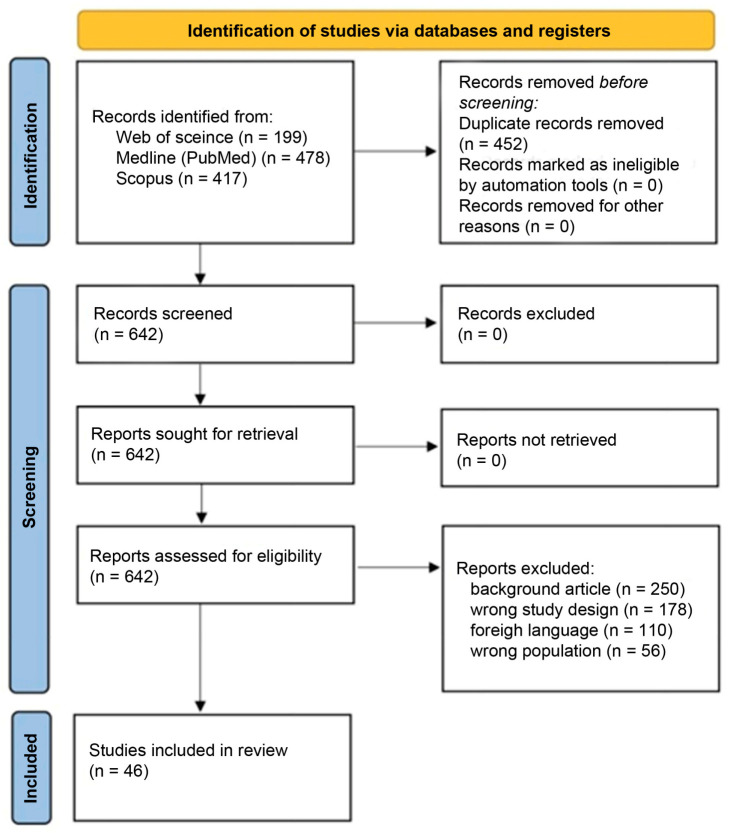
Flowchart of search process.

The systematic search identified 1094 records from Medline (PubMed), Web of Science, and Scopus up to 21 January 2025. After duplicate removal, 642 articles underwent title and abstract screening. Following full-text assessment, 46 studies met the inclusion criteria (Figure 1, Table S1).

Overall, 1636 patients underwent surgical reconstruction for post-burn neck contractures. The pooled mean age was 27.7 years, while individual study means ranged from 18.7 to 41.1 years. The study sample size ranged from 10 to 150 patients. Except for the study by Sever et al., which included only male patients, all studies involved both sexes [29]. None of the included studies evaluated direct closure, laser therapy, or stem cell-based treatment in relation to functional outcomes. The main characteristics of the included studies are summarized in Table 1.

Functional outcomes were reported in 34 studies, aesthetic outcomes in 20 studies, complications in 45 studies, recontracture in 26 studies, and disfiguring scarring in 18 studies. Secondary reconstruction following previous unsuccessful surgery was reported in six studies [21,30,31,32,33,34].

Based on reconstructive modality, studies were categorized into three groups: split-thickness skin grafts (STSG) (2 studies), dermal substitutes combined with STSG (2 studies), and flap reconstruction (42 studies). Because of the limited number of studies evaluating skin grafts and dermal substitutes, these modalities were analyzed descriptively, whereas separate analysis of treatment outcomes was performed for flap-based reconstruction.

Most studies demonstrated a low risk of bias, while a smaller proportion showed moderate or high methodological risk (Figure S1). The most common sources of bias were unclear reporting of patient recruitment, incomplete description of baseline characteristics, heterogeneous and non-standardized outcome assessment methods, and variability in follow-up duration. Despite these limitations, the majority of studies provided adequate descriptions of the surgical procedures, clinically relevant outcomes, and postoperative complications, supporting their inclusion in the evidence synthesis.

**Table 1:** The main characteristics of included studies.

Authors	Country	Patients	Age (Years)	Treatment	Number of Stages	Tissue Expansion	Mean Follow-Up (Months)	Recurrent Cases (%)	Reintervention	Outcome Assessment
Ali et al. [8]	Pakistan	28	28.6	Regional flap	1	No	3			
Angrigiani et al. 2017 [32]	Argentina	150	27.3	Distant flap	1	No	183.6	7.1	Yes, flap trimming	Objectively
Angrigiani et al. 1994 [35]	Argentina	86		Distant flap	1	No			Yes, combination of interventions	Objectively
Ayhan et al. [36]	Turkey	10	30.8	Local flap + STSG	1	No	18–24	30	Yes, excision	Objectively
Bhatti et al. [37]	Pakistan	44	29.5	Regional flap	1	No	12	4.5	No	Objectively
Chen et al. [38]	China	10	32	Regional flap	2	Yes	6		No	Subjectively
Dai et al. [39]	China	24		Local flap	2	Yes	22	0		Objectively
Gao et al. [16]	China	87	30	Flap combination	2	Yes	12	0	No	Objectively
Grishkevich et al. 2010 [40]	Russia	26		Local flap	1	No	6–108	7.7	Yes, new flap	Objectively
Grishkevich et al. 2012 [41]	Russia	32		Regional flap	1	No	144		Yes, excision	Subjectively
Grishkevich et al. 2015 [42]	Russia	21		Local flap	1	No	6–108	0		Subjectively
Hafezi et al. [43]	Iran	15	19.7	Regional flap	2	No			Yes, flap trimming	
Heidekrueger et al. [19]	Germany	20	25.2	Flap combination	2	Yes	36		Yes, combination of interventions	
Hoinoiu et al. [5]	Romania	11	34.3	Integra dermal regeneration template + STSG	2	No	18			Objectively
Hyakusoku et al. [44]	Japan	24	31.3	Regional flap	1	No	12–84		Yes, flap trimming	Subjectively
Ismail et al. [45]	Egypt	20	25.5	Regional flap	1	No	9.8	10	Yes, combination of interventions	
Jahanabadi et al. [46]	Iran	50	19.2	Local flap + STSG	1	No	12	2	Yes (no data)	Objectively
Karacaoglan et al. [47]	Turkey	12		Regional flap	2	Yes		16.6	Yes, Z plasty	
Li et al. [48]	China	15	29	Regional flap	2	Yes	12	0	No	Objectively
Loghmani et al. [33]	Iran	41	24.6	Regional flap	1	No		0	Yes (no data)	Subjectively
Luo et al. [49]	China	24		Flap combination	1	No	4–60	8.3		Objectively
Ma et al. [50]	China	66		Regional flap	1	No	6			Subjectively
Mody et al. [14]	India	22	28.2	Local flap or STSG	1	No	6	13.6		Objectively
Mun et al. [34]	South Korea	12	34.3	Distant flap	1	No	18.9	0	Yes, combination of interventions	Objectively
Nath et al. [51]	Zambia	37	23	Split-thickness skin graft	1	No	3–36	16.2	Yes	Objectively
Pallua et al. [21]	Germany	21	23.3	Flap combination	1	No	27.5	0	Yes, flap trimming	Objectively
Parrett et al. [52]	USA	32	31	Distant flap	2	Yes	12		Yes, flap trimming	
Perera et al. [53]	Sri Lanka	96	39	Split-thickness skin graft	1	No	16			Objectively
Rashid et al. [54]	Pakistan	27	32	Regional flap	1	No	22	0	Yes, Z plasty	Objectively
Saaiq et al. [55]	Pakistan	30	23.7	Regional flap	1	No	12	0		Objectively
Sarkar et al. [56]	India	11	31.9	Distant flap	1	No	66	0	No	Objectively
Seo et al. [9]	South Korea	28	32.4	AlloDerm/matriderm + STSG	1	No	16			Objectively
Sever et al. [31]	Turkey	10	21	Regional flap	2	Yes	8	0	No	
Song et al. [57]	China	12	18.7	Distant flap	3	Yes	12	16.6	Yes, combination of interventions	Objectively
Tsai et al. [20]	Taiwan	40	41.1	Distant flap	1	No	11		Yes, flap trimming	Objectively
Vinh et al. 2007 [58]	Vietnam	30	33.8	Regional flap	1	No				
Vinh et al. 2009 [59]	Vietnam	101		Regional flap	1	No		0		Subjectively
Vinh et al. 2015 [60]	Vietnam	17	26.9	Distant flap	1	No				Subjectively
Vinh et al. 2018 [61]	Vietnam	82	35	Local flap	1	No	24			Subjectively
Wang et al. 2014 [62]	China	15	21.3	Distant flap	2	Yes	12–60	0	No	Objectively
Wang et al. 2016 [63]	China	12	32.8	Regional flap	2	Yes	6–108	0		Objectively
Wang et al. 2006 [64]	China	25		Regional flap	2	Yea			No	Subjectively
Wang et al. 2012 [65]	China	68	28	Local flap	1	No	12–120	0	Yes, Z plasty	Objectively
Xie et al. [66]	China	24		Regional flap	3	Yes	10	0		
Yang et al. [67]	China	18		Regional flap	2	Yes	12			
Zhang et al. [68]	China	50	25	Flap combination	1	No	12			Objectively

### 3.2. Split-Thickness Skin Grafts

Two studies evaluated STSG reconstruction for post-burn neck contractures [67,68]. Perera et al. [53] reported restoration of the physiological neck range of motion in all patients, with a complication rate of 12.5%, primarily related to partial graft loss and hematoma. Nath et al. [51] reported satisfactory functional and aesthetic outcomes in 80% of patients, while recontracture occurred in 16% during follow-up.

### 3.3. Dermal Substitutes and Split-Thickness Skin Grafts

Two studies evaluated dermal substitutes combined with STSG [5,9]. Seo et al. [9] used AlloDerm^®^ and MatriDerm^®^ in 28 patients, reporting functional and aesthetic improvement in 64.3% of cases, with a complication rate of 39.3%. Hoinoiu et al. [5] used Integra^®^ in 11 patients and reported functional recovery in 81.8% of patients, with infection occurring in 9.1% of cases.

### 3.4. Flap Reconstruction

A total of 1464 patients from 42 studies underwent flap-based reconstruction [8,14,16,19,20,21,29,30,31,32,33,35,36,37,38,39,40,41,42,43,44,45,46,47,48,49,50,51,52,53,54,55,56,57,58,59,60,61,64,65]. Studies were categorized into four reconstructive groups: local flaps with random-pattern vascularization, regional flaps with axial vascularization, distant free flaps, and combined reconstructive techniques involving multiple flap types or flaps combined with STSG.

**Figure 2:**
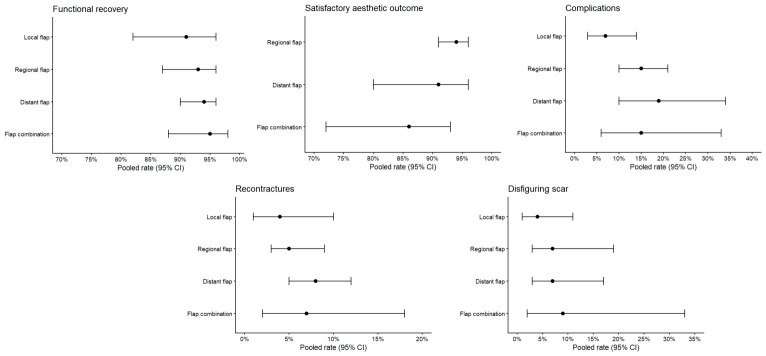
A comparative graphical representation of pooled outcome rates across flap subtypes, including functional recovery, aesthetic satisfaction, complications, recontracture and disfiguring scar rates.

To facilitate descriptive comparison across flap subtypes, pooled outcome rates for functional recovery, aesthetic satisfaction, complications, recontracture, and disfiguring scar formation are summarized in Figure 2, while detailed results for each outcome are presented in the following subsections.

#### 3.4.1. Functional Outcome

**Figure 3:**
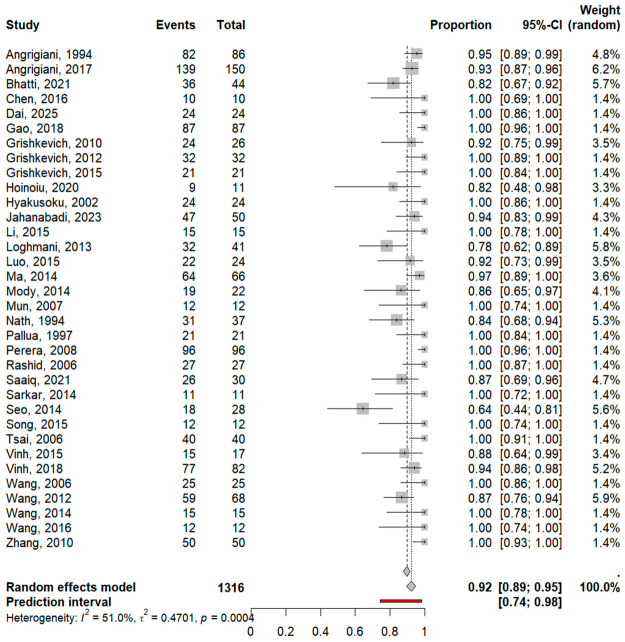
Functional outcome after post-burn neck contracture reconstruction.

Functional outcomes were assessed in 34 studies, including 1316 patients. The pooled rate of functional recovery following reconstruction was 92% (95% CI: 89–94%) (Figure 3). Moderate heterogeneity was observed (I^2^ = 51%, *p* < 0.001). Funnel plot analysis demonstrated publication bias, although influence analysis did not identify a single dominant outlier (Egger’s test *p* < 0.001, Figures S2 and S3).

Among patients reconstructed with flaps, the pooled functional success rate was 93% (95% CI: 90–95%). Additional analyses demonstrated comparable functional outcomes across flap categories, ranging from 91% for local flaps to 95% for combined reconstructive techniques (Table 2).

#### 3.4.2. Aesthetic Outcome

**Figure 4:**
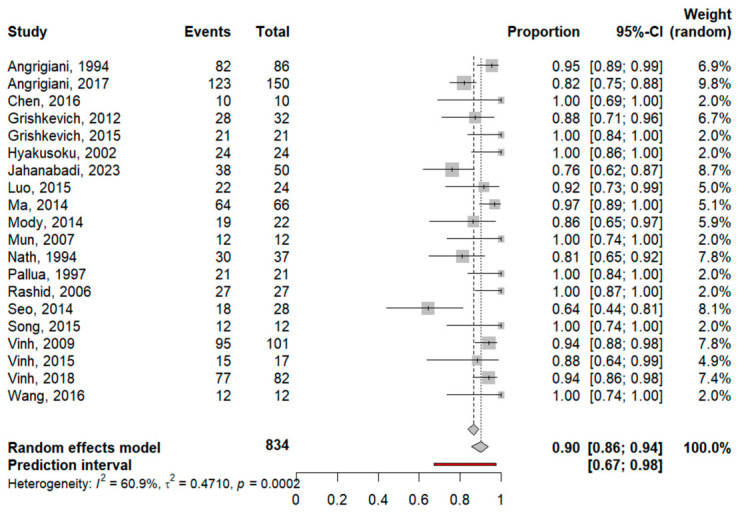
Aesthetic outcome after post-burn neck contracture reconstruction.

Aesthetic outcomes were reported in 20 studies, including 834 patients. The pooled rate of satisfactory aesthetic outcome was 90% (95% CI: 86–94%) (Figure 4). Moderate heterogeneity and publication bias were identified (Egger’s test for funnel plot asymmetry *p* = 0.002, Figures S4 and S5).

Flap reconstruction achieved a pooled aesthetic success rate of 92% (95% CI: 87–95%). Regional flaps demonstrated the highest pooled aesthetic success rate (94%), whereas combined reconstructive techniques showed the lowest rate (86%) (Table 2).

#### 3.4.3. Complications

**Figure 5:**
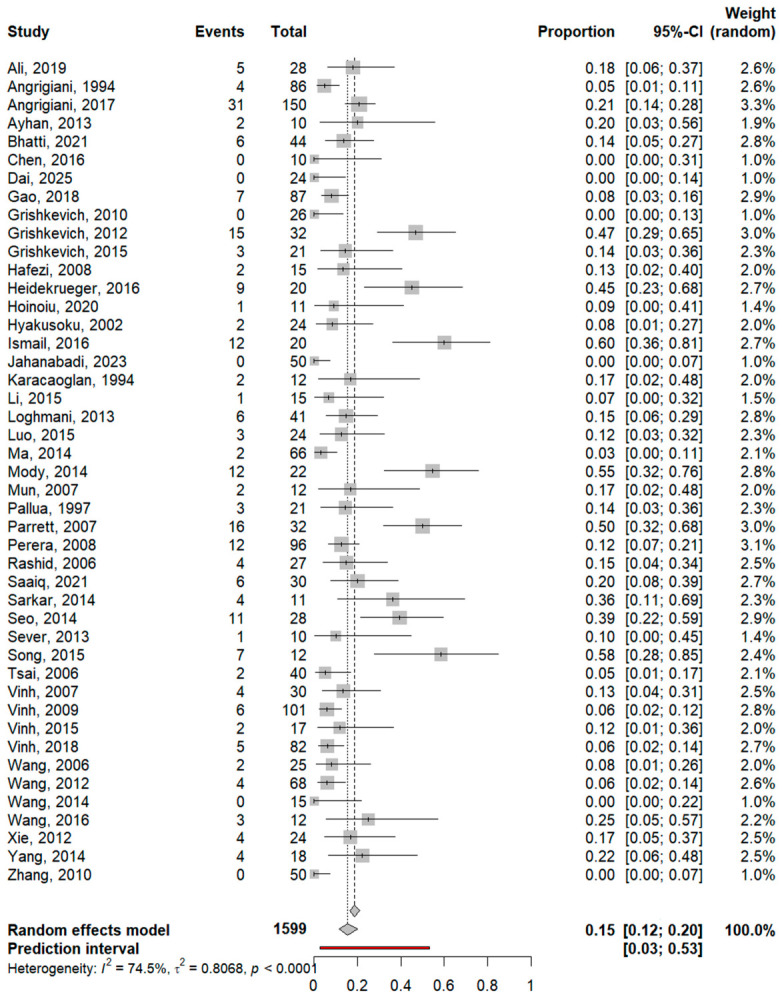
Complication rates after post-burn neck contracture reconstruction.

Complications were reported in 45 studies, including 1599 patients. The pooled complication rate after reconstruction was 15% (95% CI: 12–20%) (Figure 5). Significant heterogeneity was observed across studies (I^2^ = 74.5%, *p* < 0.001), accompanied by evidence of publication bias (Figures S6 and S7).

Among flap-based procedures, the pooled complication rate was also 15% (95% CI: 11–20%). Local flaps demonstrated the lowest complication rate (7%), whereas distant free flaps showed the highest rate (19%) (Table 2). The highest variability regarding the complication rate was recorded in patients treated with distant flap (I^2^ = 82%) and flap combinations (I^2^ = 82%), followed by regional flap (I^2^ = 68,7%), while the lowest variability was recorded with local flap approach (Table 2).

#### 3.4.4. Recontracture

**Figure 6:**
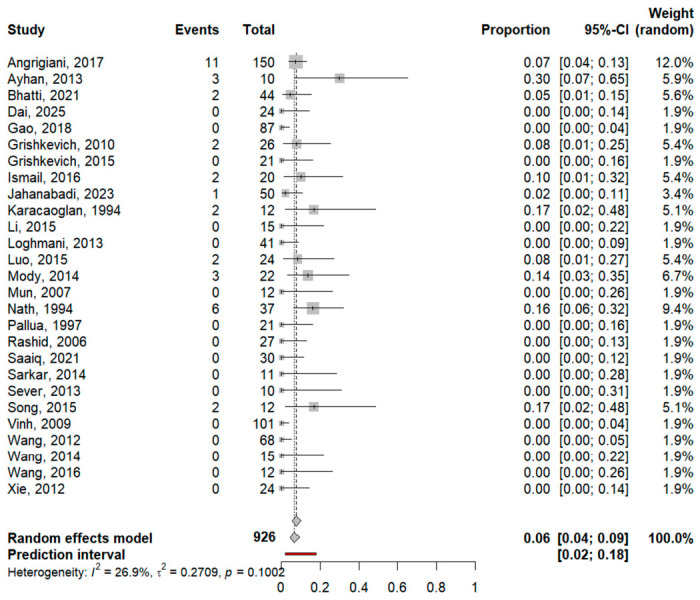
Recontracture rates after post-burn neck contracture reconstruction.

Recontracture was assessed in 27 studies, including 926 patients. The pooled recontracture rate following reconstruction was 6% (95% CI: 4–9%) (Figure 6). Low-to-moderate heterogeneity was observed (I^2^ = 26.9%, *p* = 0.10). Sensitivity analysis excluding influential studies did not significantly alter pooled estimates (Figures S8–S10; Table S2).

Among flap-based reconstructions, the pooled recontracture rate was 4% (95% CI: 4–9%). Local flaps demonstrated the lowest incidence of recontracture (4%), whereas distant free flaps demonstrated the highest incidence (8%) (Table 2).

#### 3.4.5. Disfiguring Scars

**Figure 7:**
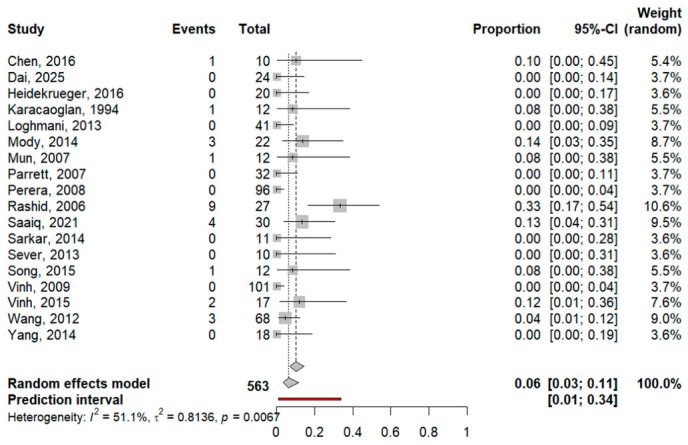
Disfiguring scars after post-burn neck contracture reconstruction.

Disfiguring scarring was evaluated in 18 studies, including 563 patients. The pooled rate of disfiguring scars was 6% (95% CI: 3–11%) (Figure 7). Moderate heterogeneity and publication bias were observed (Figures S11 and S12).

Among flap-based procedures, the pooled incidence of disfiguring scars was 7% (95% CI: 4–13%). Local flaps demonstrated the lowest incidence of disfiguring scarring (4%), whereas combined reconstructive techniques demonstrated the highest rate (9%) (Table 2).

#### 3.4.6. Reintervention

Reintervention was inconsistently reported across the included studies and was therefore not suitable for quantitative synthesis (Table 1). When reported, secondary procedures most commonly included flap trimming, scar revision, Z-plasty, or additional flap reconstruction. These interventions were generally performed to optimize contour, improve functional or aesthetic outcomes, or address residual contracture rather than to treat major postoperative complications. Owing to heterogeneous reporting and the absence of standardized definitions, reintervention was summarized descriptively only.

The certainty of evidence assessed using the GRADE approach is summarized in Table S3. Overall, the certainty of evidence was rated as low for all outcomes because all included studies were observational in design. Additional concerns included risk of bias, publication bias and heterogeneity across studies.

## 4. Discussion

This systematic review and meta-analysis demonstrated that flap-based reconstruction remains the cornerstone of surgical management for post-burn neck contractures. Despite the considerable evolution of reconstructive techniques over recent decades, the pooled evidence suggests that flap-based reconstruction may provide durable long-term restoration of both function and appearance. The present findings support the principle that durable correction of post-burn neck deformities requires not only release of the contracture itself but also resurfacing with tissue capable of maintaining elasticity and resisting secondary contraction. Across the included studies, reconstruction was associated with high rates of functional improvement and satisfactory aesthetic outcomes, while the incidence of recontracture and disfiguring scarring remained relatively low. These findings are clinically important because post-burn neck contractures affect far more than neck mobility alone. The resulting deformities may impair airway access, oral competence, visual orientation, and daily activities, while simultaneously producing substantial psychosocial burden due to visible facial and cervical disfigurement [4,6,7].

Although the pooled estimates demonstrated favorable functional and aesthetic outcomes, these findings should be interpreted with appropriate caution. The certainty of the available evidence remains limited because all included studies were observational, predominantly retrospective case series, with important methodological limitations, heterogeneous patient populations, and considerable variability in outcome assessment, including both objective measurements and subjective surgeon- or patient-reported evaluations. Consequently, the pooled estimates should be interpreted as a synthesis of the currently available evidence rather than definitive comparative evidence regarding the superiority of individual reconstructive techniques. This methodological approach reflects the current state of the literature, in which outcome reporting remains heterogeneous and no consensus exists regarding standardized assessment of functional or aesthetic recovery after post-burn neck reconstruction.

The long-term consequences of post-burn neck contractures extend beyond physical impairment and represent an important source of disability among burn survivors. Advances in acute burn care have significantly improved survival following severe burn injury, shifting attention toward long-term rehabilitation and restoration of quality of life. Consequently, reconstructive surgery is increasingly evaluated not only according to technical success but also according to its ability to restore function, appearance, social participation, and psychological well-being. Although patient-reported outcomes were rarely reported in the studies included in this review, the consistently high rates of functional and aesthetic improvement suggest that successful reconstruction may contribute substantially to long-term recovery and reintegration into society.

One of the most important findings of the present study is that flap-based reconstruction consistently outperformed skin grafting and dermal substitute-based approaches in terms of durability. While satisfactory functional outcomes were achieved across multiple reconstructive modalities, flap reconstruction was associated with lower rates of recontracture and more predictable long-term results. This observation is biologically plausible because flaps provide vascularized tissue with superior elasticity, thickness, and resistance to secondary contraction compared with skin grafts. In highly mobile anatomical regions such as the neck, maintenance of tissue pliability is essential for preserving neck extension and preventing recurrence of deformity over time.

Although flap reconstruction demonstrated favorable overall outcomes, important differences emerged among flap categories. Local, regional, and distant free flaps all achieved high rates of functional recovery, indicating that successful contracture release can be accomplished using different reconstructive approaches when appropriate patient selection is applied. However, the balance between functional restoration, aesthetic outcome, operative complexity, and complication risk varied considerably between reconstructive strategies.

Local flaps demonstrated excellent safety profiles, with the lowest pooled rates of complications, recontracture, and disfiguring scarring. Their favorable outcomes likely reflect careful patient selection, as these techniques were predominantly utilized in patients with relatively limited contractures and preserved surrounding healthy tissue [14,46,69,70,71,72]. The principal advantages of local flaps include technical simplicity, shorter operative time, minimal donor-site morbidity, and excellent tissue match with respect to color, texture, and thickness [42,69,70,71,72,73]. In addition, preservation of regional vascular anatomy and avoidance of microsurgical procedures make these techniques particularly attractive in selected patients. However, the amount of transferable tissue remains limited, restricting their applicability in extensive deformities involving large neck surface areas [8,71,72]. Consequently, local flaps should be viewed as valuable options for mild-to-moderate contractures rather than universal reconstructive solutions.

Based on the pooled descriptive analyses, regional flaps appeared to provide a favorable balance between functional recovery, aesthetic outcome, and complication rates. However, because these observations are derived from indirect comparisons across heterogeneous observational studies rather than head-to-head comparative trials, they should be interpreted as hypothesis-generating rather than definitive evidence of superiority. These procedures achieved excellent functional outcomes while simultaneously producing the highest pooled aesthetic success rates and maintaining acceptable complication rates. Among the various regional flap techniques, supraclavicular artery-based flaps were particularly prominent throughout the literature [8,31,33,37,54,55,58,59]. Their popularity is understandable because they combine several characteristics that are particularly advantageous in neck reconstruction, including reliable vascularity, favorable skin color and texture match, relative technical simplicity, and close anatomical proximity to the defect. Unlike bulkier musculocutaneous flaps, fasciocutaneous regional flaps preserve neck contour and flexibility more effectively, contributing to restoration of a natural cervicomental angle and more physiological neck movement [8,31,71,74].

The predominance of supraclavicular artery-based flaps within the contemporary literature is unlikely to be coincidental. From a reconstructive standpoint, these flaps address several of the principal challenges encountered in neck resurfacing simultaneously. They provide thin and pliable tissue, allow restoration of the cervicomental angle, avoid the complexity of microsurgical transfer, and generally achieve superior color and texture matching compared with distant donor sites. For these reasons, their increasing popularity observed across studies likely reflects genuine clinical advantages rather than simple surgeon preference.

An additional advantage of regional flaps is their ability to resurface relatively large defects without requiring microsurgical anastomosis [33]. This reduces operative complexity, shortens surgical time, and facilitates broader applicability across reconstructive centers. Furthermore, the frequent use of tissue expansion in several studies reflects ongoing efforts to maximize flap dimensions while minimizing donor-site morbidity [31,38,47,63,64,66,67]. Taken together, these findings suggest that regional fasciocutaneous flaps, particularly supraclavicular artery-based flaps, currently represent one of the most versatile reconstructive options for moderate and severe post-burn neck contractures.

Distant free flaps were primarily used in patients with extensive deformities, severe surrounding scarring, recurrent contractures, or insufficient regional donor-site availability [21,32,34,56]. Within this challenging patient population, free tissue transfer achieved excellent restoration of neck mobility, emphasizing its indispensable role in advanced reconstructive surgery. Nevertheless, this functional benefit was accompanied by the highest pooled complication rates observed in the present analysis.

Importantly, the higher complication rates observed following free flap reconstruction should not necessarily be interpreted as evidence of inferior performance. In most studies, free tissue transfer was reserved for the most severe and challenging deformities, frequently involving extensive scarring, recurrent contractures, or limited regional reconstructive options. Consequently, the observed complication profile may partly reflect the complexity of the underlying clinical scenarios rather than the reconstructive method itself.

Microsurgical reconstruction in burn patients remains technically demanding because recipient vessels may be affected by previous injury, scarring, or multiple prior interventions. In addition, prolonged operative time and the frequent need for secondary contouring procedures may further increase overall treatment complexity [47,72]. Aesthetic integration also represents an important consideration when evaluating free flap reconstruction. Donor-site tissues obtained from the thigh, scapular region, or other distant anatomical locations may differ substantially from neck skin in thickness, texture, pigmentation, and hair-bearing characteristics [34,56]. Although modern perforator-based and super-thin flap techniques have improved contouring and flexibility [56,60,61], aesthetic outcomes generally remained somewhat inferior to those achieved with regional flaps. Therefore, free tissue transfer should not be viewed as a superior reconstructive strategy for all patients but rather as a highly valuable option for selected individuals with severe or recurrent deformities in whom local and regional alternatives are insufficient.

The subgroup comprising combined reconstructive approaches demonstrated another important principle of modern burn reconstruction. In many patients, optimal outcomes cannot be achieved through a single reconstructive technique. Instead, successful reconstruction frequently requires individualized combinations of local flaps, regional flaps, free flaps, skin grafts, tissue expansion, or staged procedures [16,19,21,49,68]. Such approaches acknowledge the considerable heterogeneity of post-burn neck deformities and emphasize the importance of tailoring reconstruction to defect severity, tissue quality, previous interventions, and donor-site availability. Several authors highlighted that successful treatment should aim not only to restore neck extension but also to recreate the cervicomental angle and neck contour, both of which contribute substantially to overall aesthetic perception and patient satisfaction [49,68].

In contrast to flap-based reconstruction, skin grafting and dermal substitute-based approaches demonstrated less consistent outcomes and a greater tendency toward recurrence. Although split-thickness skin grafting remains widely available and technically straightforward, secondary contraction continues to represent a major limitation, particularly in highly mobile regions such as the neck [14,46,75,76]. Dermal substitutes have been introduced to address some of these shortcomings by improving elasticity and reducing contracture formation [5,77,78,79,80,81,82]. These technologies may be particularly useful in extensively burned patients with limited donor-site availability or when flap reconstruction is not feasible. However, the currently available evidence remains limited and consists primarily of small observational studies.

Nevertheless, the continued presence of skin grafting within contemporary reconstructive practice illustrates an important clinical reality. In many healthcare settings, particularly those with limited reconstructive resources, skin grafts remain more accessible than complex flap procedures. Therefore, although graft-based reconstruction may not provide outcomes comparable to flap surgery, it continues to represent an important therapeutic option for selected patients.

From a clinical perspective, the findings of this review support an individualized reconstructive algorithm rather than a single preferred technique. Patients with mild contractures and preserved adjacent healthy tissue may benefit from local flap reconstruction, which offers excellent safety and tissue matching. Moderate contractures frequently appear to be best managed with regional fasciocutaneous flaps, particularly supraclavicular artery-based flaps, which provide an optimal balance between function, aesthetics, and operative complexity. In contrast, severe or recurrent deformities often require free tissue transfer because local and regional options may not provide sufficient tissue volume or surface area. Skin grafts and dermal substitutes remain useful in selected circumstances but should generally be considered adjunctive rather than primary reconstructive modalities. Although this proposed algorithm should not be interpreted as prescriptive guidance, it may provide a practical framework for clinical decision-making until higher-quality comparative evidence becomes available.

Another important observation arising from this review is the persistent lack of standardized outcome assessment in studies evaluating post-burn neck reconstruction. Considerable variability exists in the methods used to evaluate both functional and aesthetic outcomes, ranging from objective measurements of neck range of motion and cervicomental angle to subjective surgeon-reported assessments [27,28,29,33,38,41,42,44,50,59,60,61]. Such heterogeneity substantially limits direct comparison between studies and complicates evidence synthesis. Future investigations would benefit from the development of consensus-based outcome measures that combine objective functional evaluation with standardized aesthetic assessment and validated patient-reported outcome instruments.

Perhaps the most striking finding identified during this review was the near-complete absence of patient-reported outcome measures. While restoration of neck extension and reduction in recontracture rates remain important surgical objectives, these parameters alone cannot fully capture reconstructive success from the patient’s perspective. The visible nature of neck deformities means that appearance, self-confidence, social interaction, and quality of life frequently influence overall satisfaction as much as functional recovery. As burn care increasingly adopts patient-centered models of outcome assessment, the incorporation of validated quality-of-life instruments should become a priority in future reconstructive studies.

Several limitations should be acknowledged. Most included studies were retrospective case series without control groups, resulting in an overall low level of evidence. No randomized controlled trials were identified, limiting the ability to draw causal inferences regarding the comparative effectiveness of reconstructive techniques. Considerable heterogeneity existed with respect to patient populations, reconstructive techniques, follow-up duration, and outcome assessment methods. In addition, a substantial proportion of the included studies originated from a limited number of countries and specialized burn centers, which may limit the generalizability of the findings to other healthcare settings and populations.

The major limitation was the inconsistent reporting of baseline clinical characteristics across the included studies. Contracture severity was described using heterogeneous classification systems, qualitative assessments, or was not reported, precluding meaningful study-level stratification or subgroup analyses based on this variable.

An additional limitation relates to the heterogeneity of outcome assessment across the included studies. Functional recovery and aesthetic outcomes were evaluated using a variety of objective measurements and subjective surgeon- or patient-reported assessments because no standardized evaluation system currently exists for post-burn neck contracture reconstruction. Consequently, pooling these outcomes may have introduced measurement bias and should be interpreted with appropriate caution. Nevertheless, inclusion of both objective and subjective assessments allowed a more comprehensive synthesis of the currently available literature while reflecting contemporary clinical practice. Some included studies enrolled mixed-age populations; however, detailed age distributions were inconsistently reported, precluding subgroup analyses based on age.

Because the available evidence consisted almost exclusively of single-arm observational studies, direct statistical comparisons between reconstructive techniques were not feasible. Consequently, differences observed across reconstructive modalities should be regarded as descriptive rather than confirmatory. Publication bias was identified in several analyses, reflecting the tendency of reconstructive surgical literature to preferentially report favorable outcomes from specialized centers. In addition, evidence regarding dermal substitutes, regenerative approaches, and emerging technologies remains limited. Finally, the absence of standardized patient-reported outcome measures and cost-effectiveness analyses restricted the ability to evaluate broader dimensions of reconstructive success.

Future advances in burn reconstruction may also arise from regenerative approaches, including fat grafting, stromal vascular fraction, adipose-derived stem cells, tissue-engineered skin substitutes, and bioprinting. Although these technologies have shown encouraging preliminary results in tissue regeneration and scar modulation, their role in the management of mature post-burn neck contractures remains to be established through well-designed clinical studies.

Taken together, the available evidence suggests that successful reconstruction of post-burn neck contractures depends less on identification of a single superior technique and more on appropriate matching of reconstructive strategy to defect characteristics and patient-specific factors. Although flap-based reconstruction remains the foundation of contemporary management, different flap categories appear to occupy complementary rather than competing roles within the reconstructive ladder. Future multicenter prospective studies incorporating standardized functional assessment, aesthetic evaluation, and patient-reported outcomes are needed to refine treatment algorithms and further improve long-term outcomes for burn survivors.

## 5. Conclusions

Flap-based reconstruction remains the cornerstone of surgical management for post-burn neck contractures, providing high rates of functional recovery and satisfactory aesthetic outcomes with low rates of recontracture. Based on the available evidence, regional flaps appeared to provide a favorable risk–benefit profile, combining high rates of functional and aesthetic improvement with acceptable complication rates. However, these observations are based on indirect comparisons across heterogeneous observational studies and should not be interpreted as evidence of superiority over other reconstructive techniques. Future prospective multicenter studies incorporating standardized outcome measures and patient-reported outcomes are needed to establish stronger evidence-based reconstructive algorithms for post-burn neck contractures.

## Figures and Tables

**Table 2:** Reporting the outcomes after surgical reconstruction of post-burn neck contracture utilizing flaps.

Outcome	Studies Reporting Outcome (Events/Population)	Poled Rate (95% CI)	*I*^2^ (*p* Value)	Reported Range	Prediction Interval
**Functional recovery**	30 (1080/1144)	93% (90–95%)	30.4% (0.060)	78–100%	74–98%
Local flap	4 (128/139)	91% (82–96%)	15% (0.317)	87–100%	
Regional flap	12 (380/408)	93% (87–96%)	49.7% (0.025)	78–100%	
Distant flap	8 (326/343)	94% (90–96%)	0% (0.833)	88–100%	
Flap combination	6 (246/254)	95% (88–98%)	32.9% (0.189)	86–100%	
**Satisfactory aesthetic outcome**	17 (681/748)	92% (87–95%)	52% (0.005)	76–100%	67–98%
Local flap	1 (21/21)	/	/	/	
Regional flap	8 (337/354)	94% (91–96%)	0% (0.681)	88–100%	
Distant flap	5 (244/277)	91% (80–96%)	57.8% (0.050)	82–100%	
Flap combination	4 (100/117)	86% (72–93%)	44.1% (0.146)	76–100%	
**Complications**	42 (203/1464)	15% (11–20%)	74.7% (<0.001)	0–60%	3–53%
Local flap	4 (7/139)	7% (3–14%)	15% (0.317)	0–14%	
Regional flap	21 (92/666)	15% (10–21%)	68.7% (<0.001)	0–60%	
Distant flap	9 (68/375)	19% (10–34%)	82% (<0.001)	0–58%	
Flap combination	8 (36/284)	15% (6–33%)	82% (<0.001)	0–55%	
**Recontractures**	26 (30/889)	6% (4–9%)	19.7% (0.184)	0–30%	2–18%
Local flap	4 (2/139)	4% (1–10%)	0% (0.424)	0–8%	
Regional flap	11 (6/336)	5% (3–9%)	0% (0.471)	0–17%	
Distant flap	5 (13/200)	8% (5–12%)	0% (0.699)	0–17%	
Flap combination	6 (9/214)	7% (2–18%)	60.2% (0.027)	0–30%	
**Disfiguring scar**	17 (25/467)	7% (4–13%)	46.7% (0.018)	0–33%	1–34%
Local flap	2 (3/92)	4% (1–11%)	0% (0.597)	0–4%	
Regional flap	8 (15/249)	7% (3–19%)	64.3% (0.006)	0–33%	
Distant flap	5 (4/84)	7% (3–17%)	0% (0.734)	0–12%	
Flap combination	2 (3/42)	9% (2–33%)	30.2% (0.231)	0–14%	

## Data Availability

The data presented in this study are available from the corresponding author upon reasonable request.

## Supplementary Materials

The following supporting information can be downloaded at: https://www.mdpi.com/article/10.3390/jcm15145583/s1, Figure S1: JBI risk of bias traffic light plot; Figure S2: Publication bias for functional outcomes; Figure S3: Influence analysis for functional outcomes.; Figure S4: Publication bias for aesthetic outcomes; Figure S5: Influence analysis for aesthetic outcomes; Figure S6: Publication bias for complications: Figure S7: Influence analysis for complications; Figure S8: Publication bias for recontracture; Figure S9: Influence analysis for recontracture; Figure S10: Meta-analysis of recontracture rates following exclusion of influential studies; Figure S11: Influence analysis for disfiguring scars; Figure S12: Publication bias for disfiguring scars; Table S1: PRISMA 2020 Checklist; Table S2: Influence analysis of studies reporting on contracture occurrence; Table S3: GRADE Summary of Findings.
