# Supplementary material for: Reconstruction of Post-Burn Neck Contractures: A Systematic Review and Meta-Analysis Comparing Surgical Techniques and Outcomes
Source: J Clin Med. 2026 Jul 16;15(14):5583. doi: 10.3390/jcm15145583 (PMC13413205; doi:10.3390/jcm15145583)

Supplementary- Figures S1-S12

Figure S1. JBI risk of bias traffic light plot.

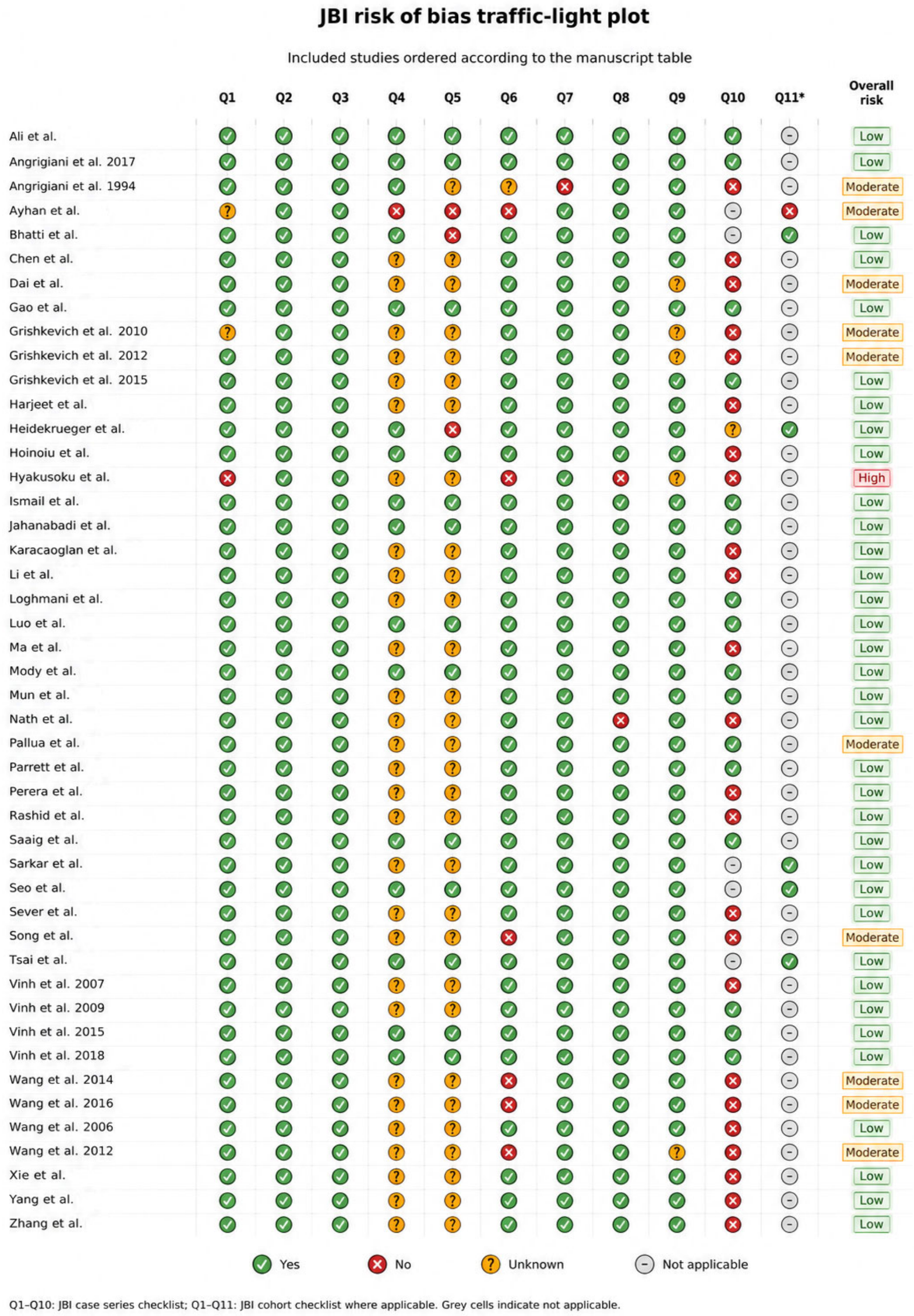

.

Figure S2. Publication bias for functional outcomes.

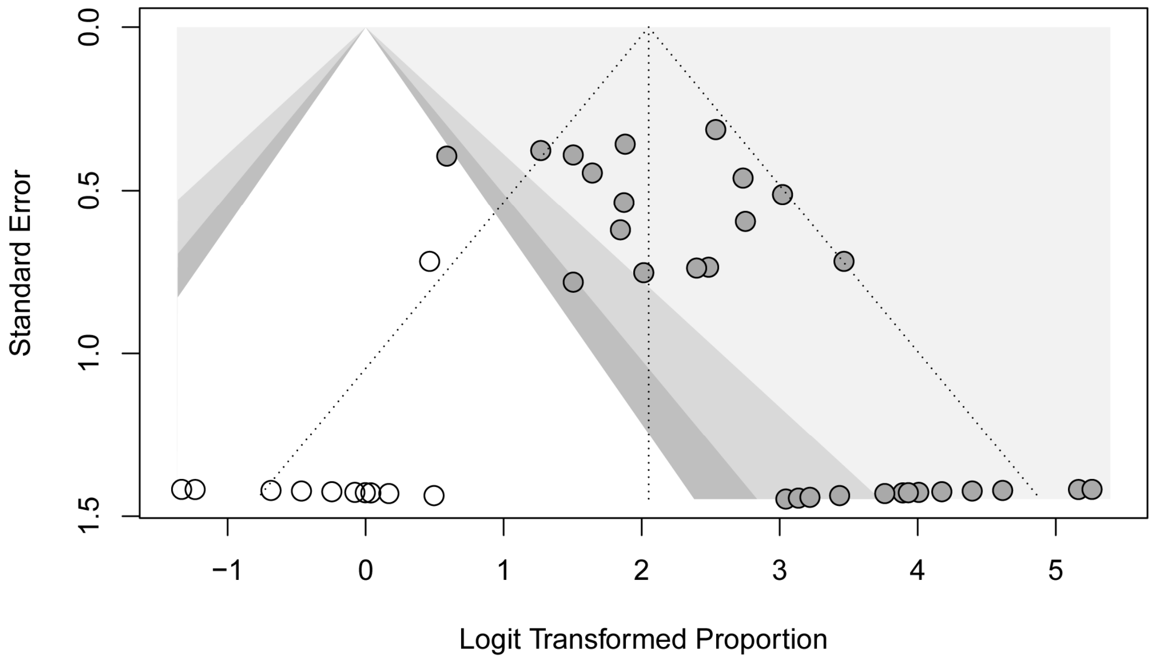

Figure S3. Influence analysis for functional outcomes.

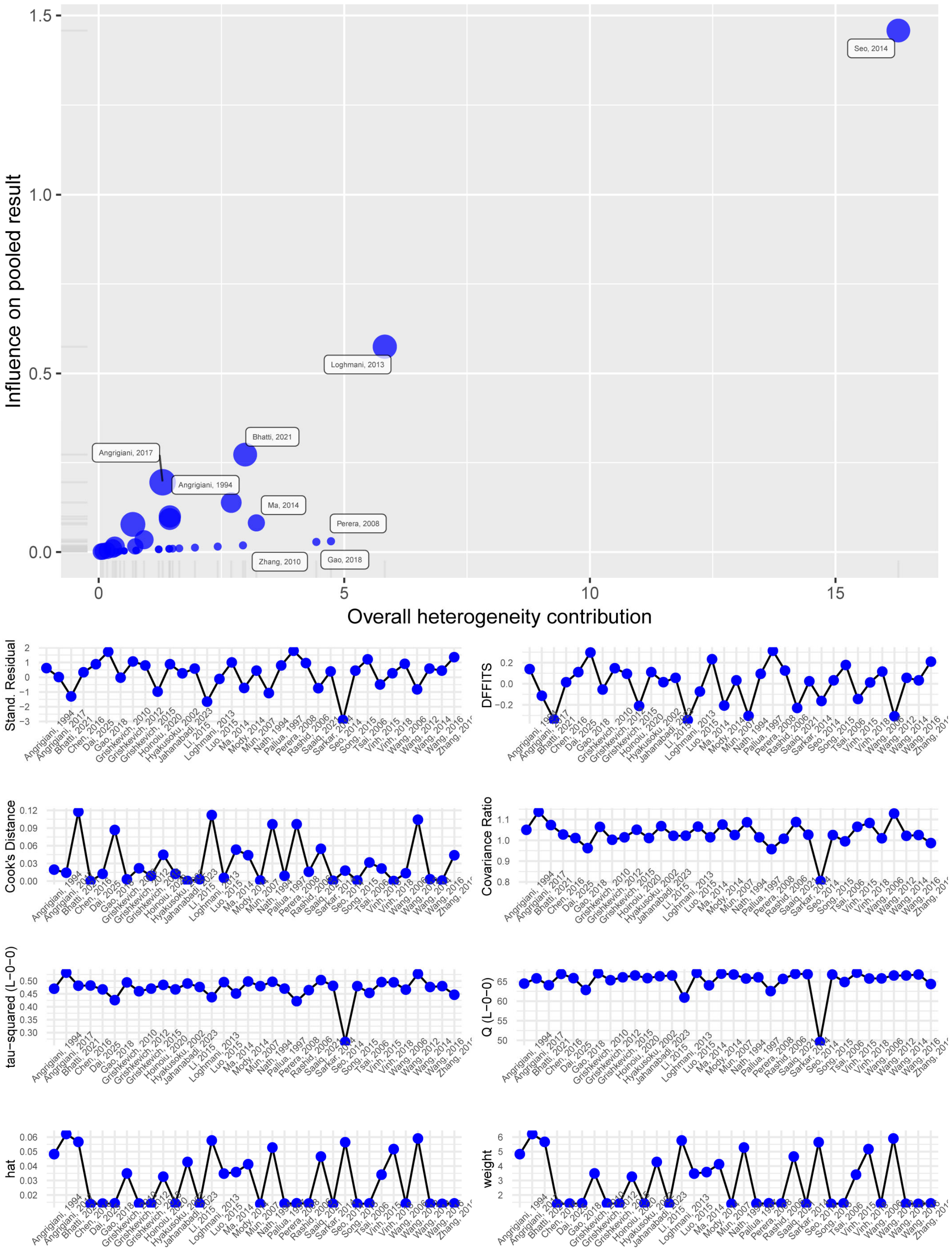

Figure S4. Publication bias for aesthetic outcomes.

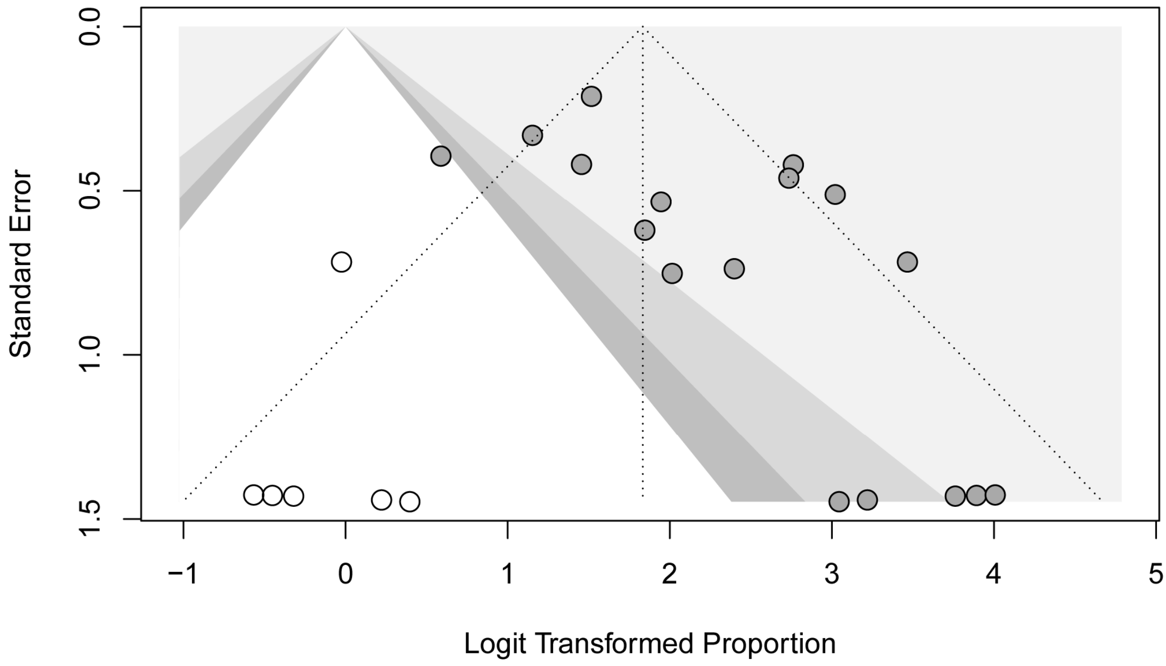

Figure S5. Influence analysis for aesthetic outcomes.

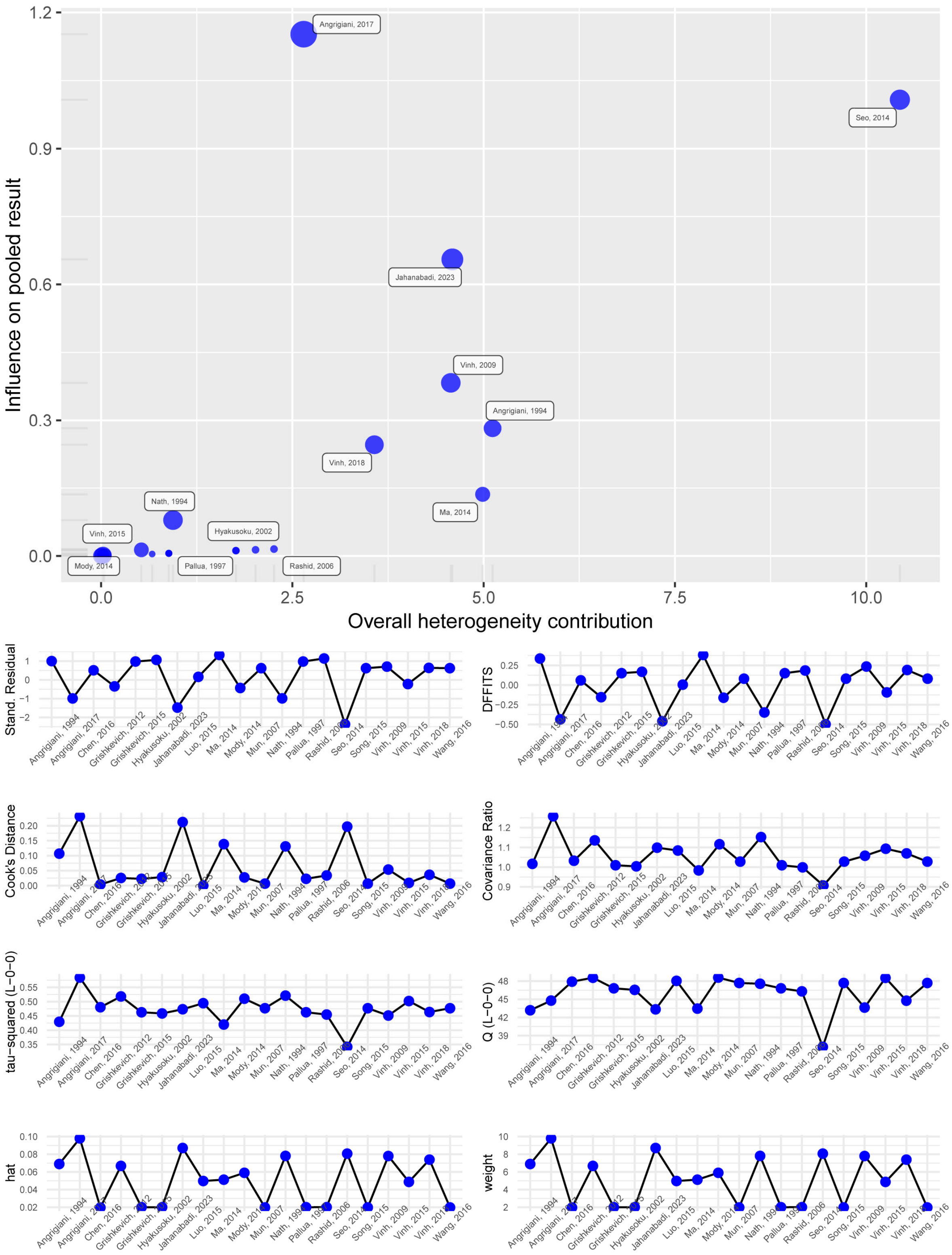

Figure S6. Publication bias for complications.

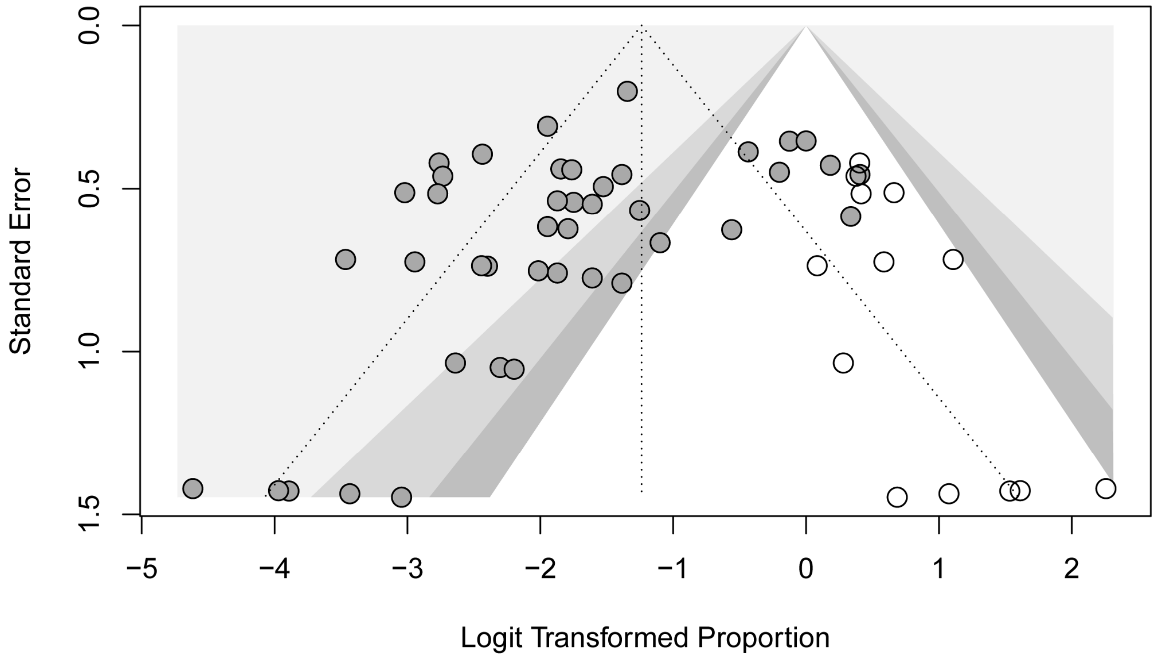

Figure S7. Influence analysis for complications.

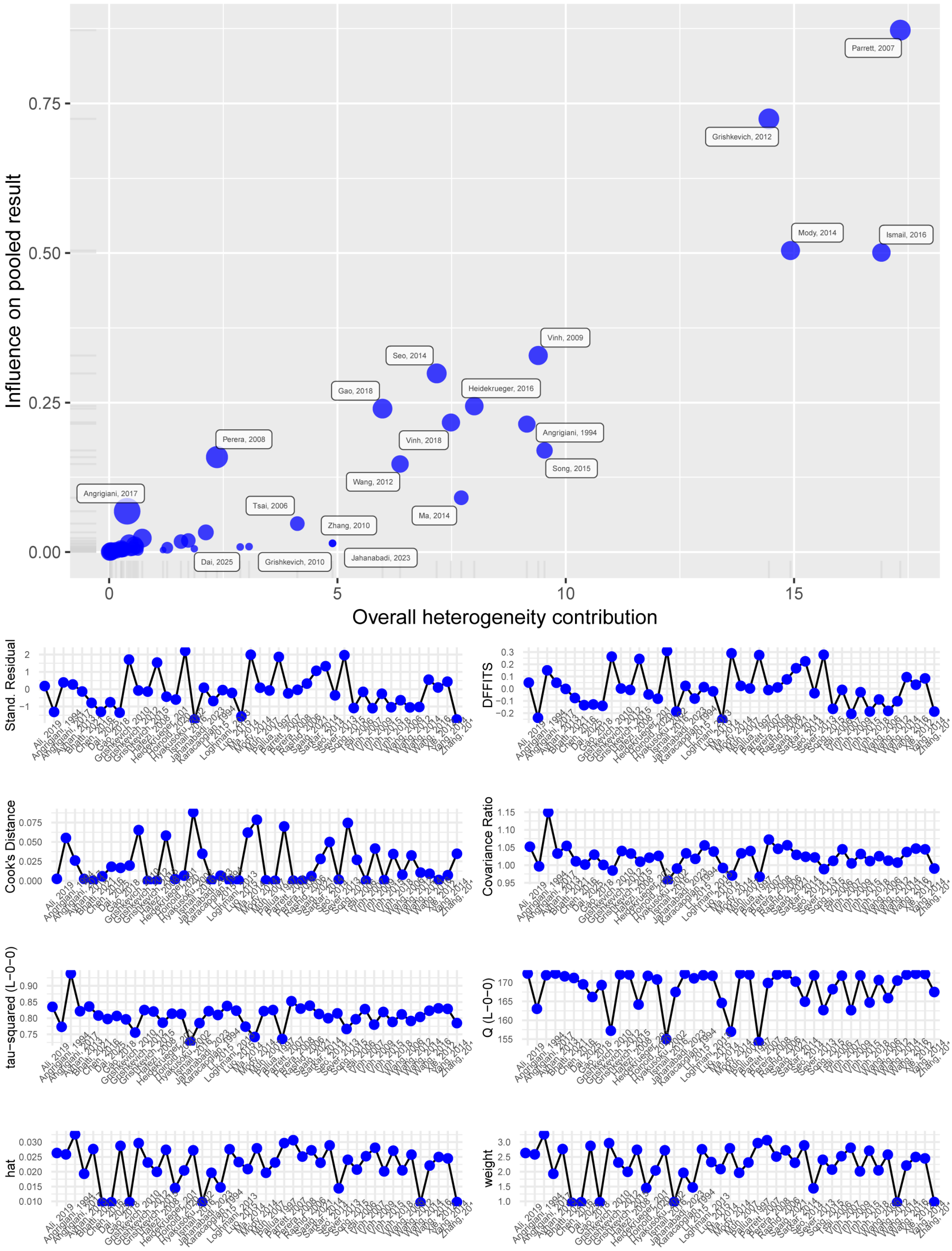

Figure S8. Publication bias for recontracture.

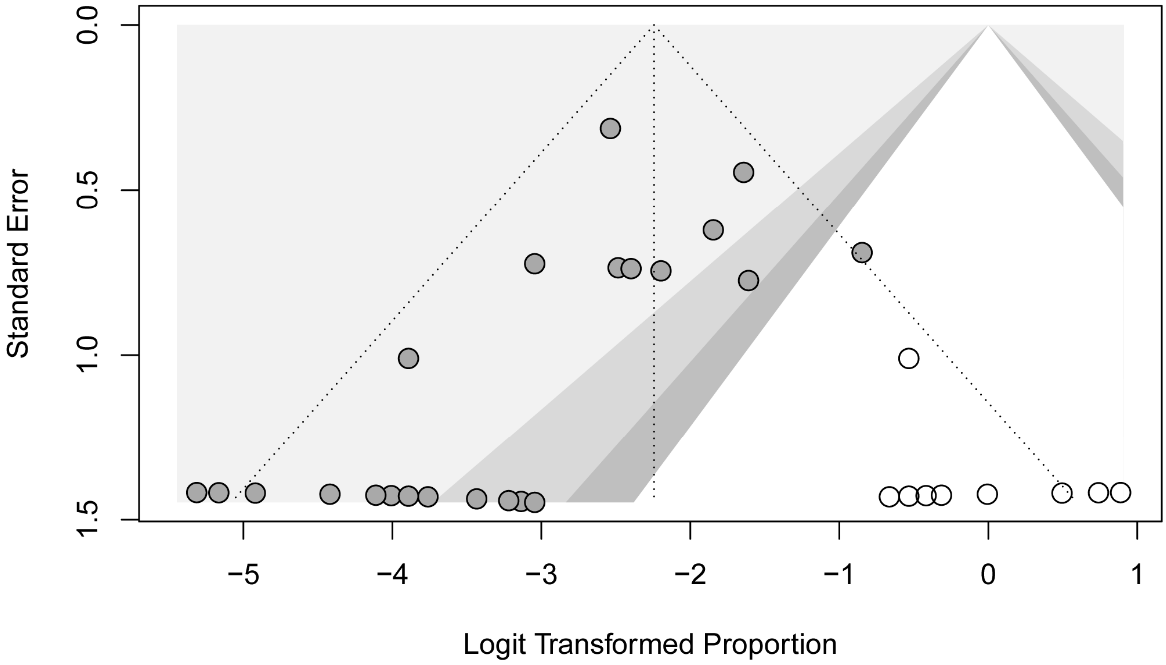

Figure S9. Influence analysis for recontracture.

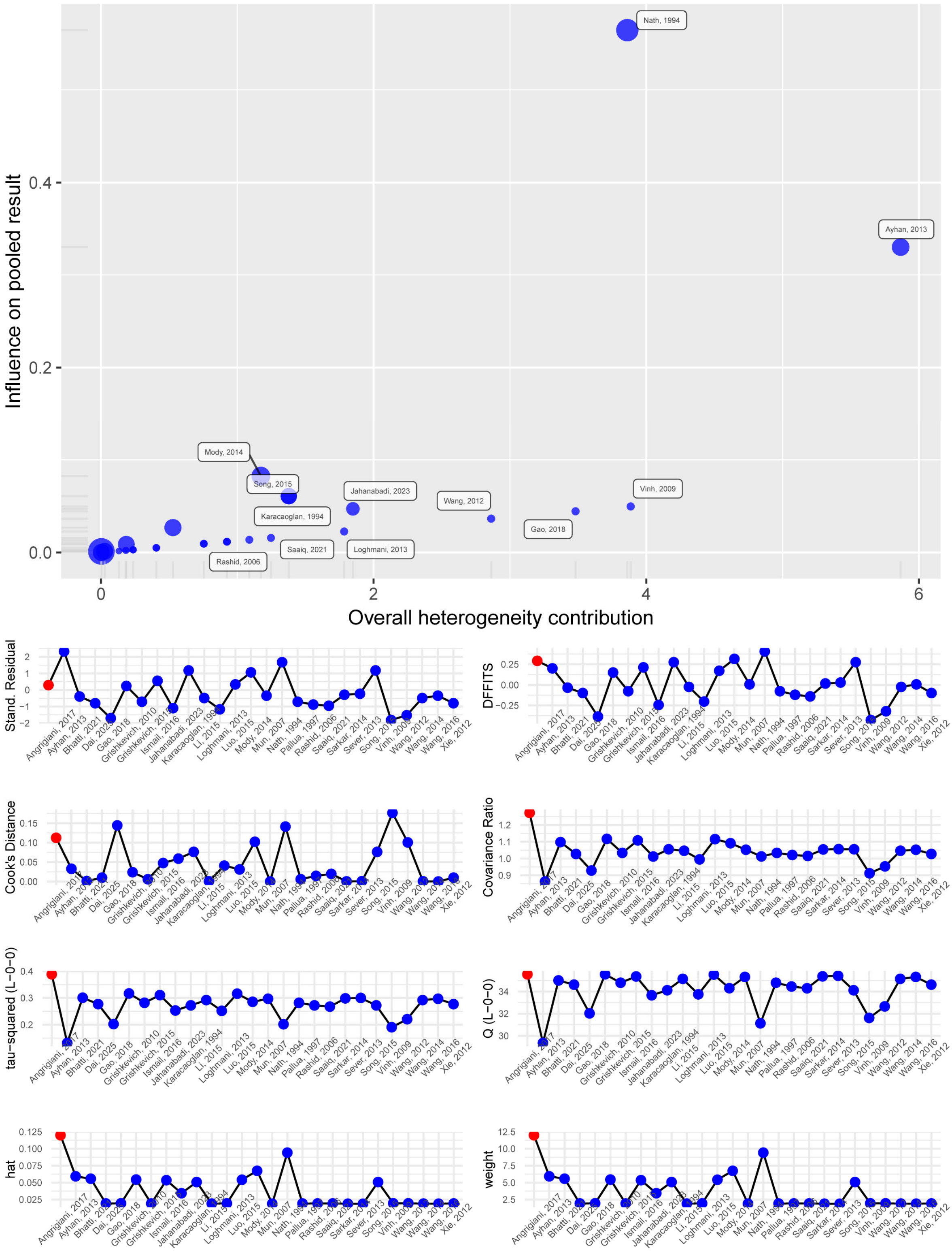

Figure S10. Meta-analysis of recontracture rates following exclusion of influential studies.

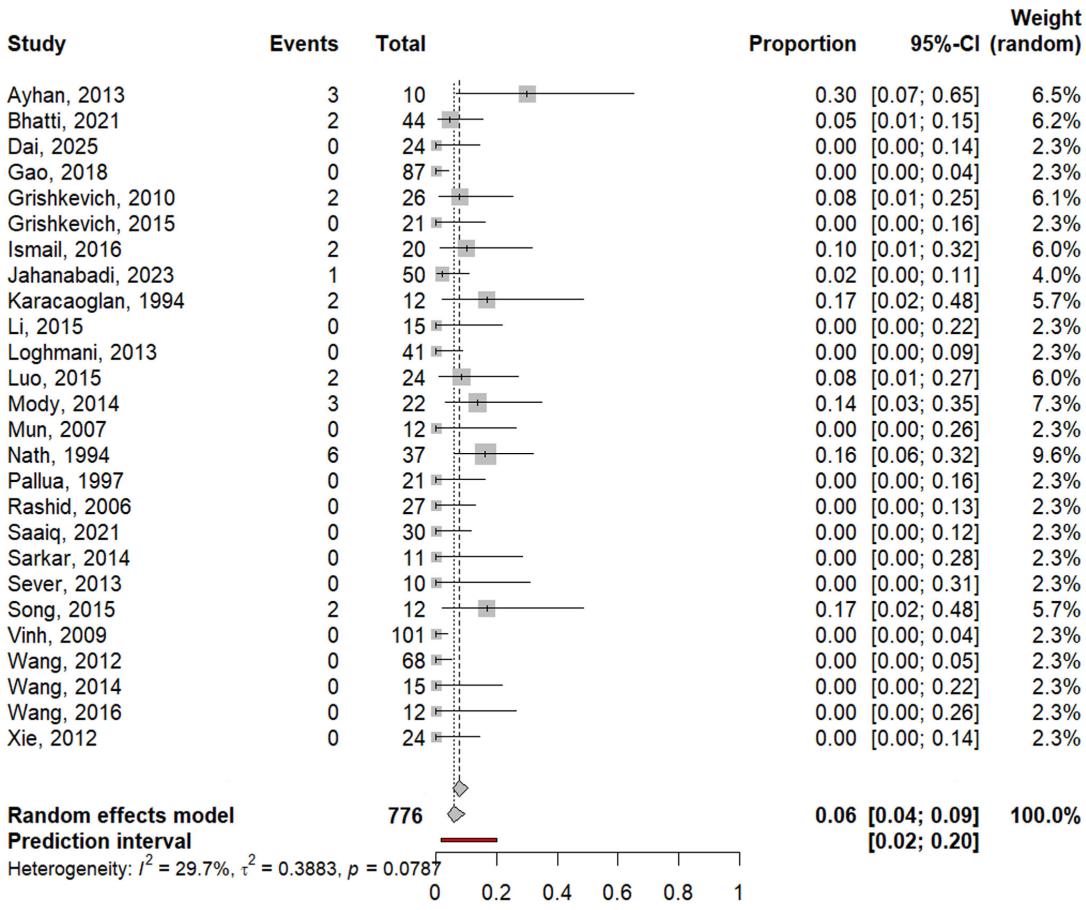

Figure S11. Influence analysis for disfiguring scars.

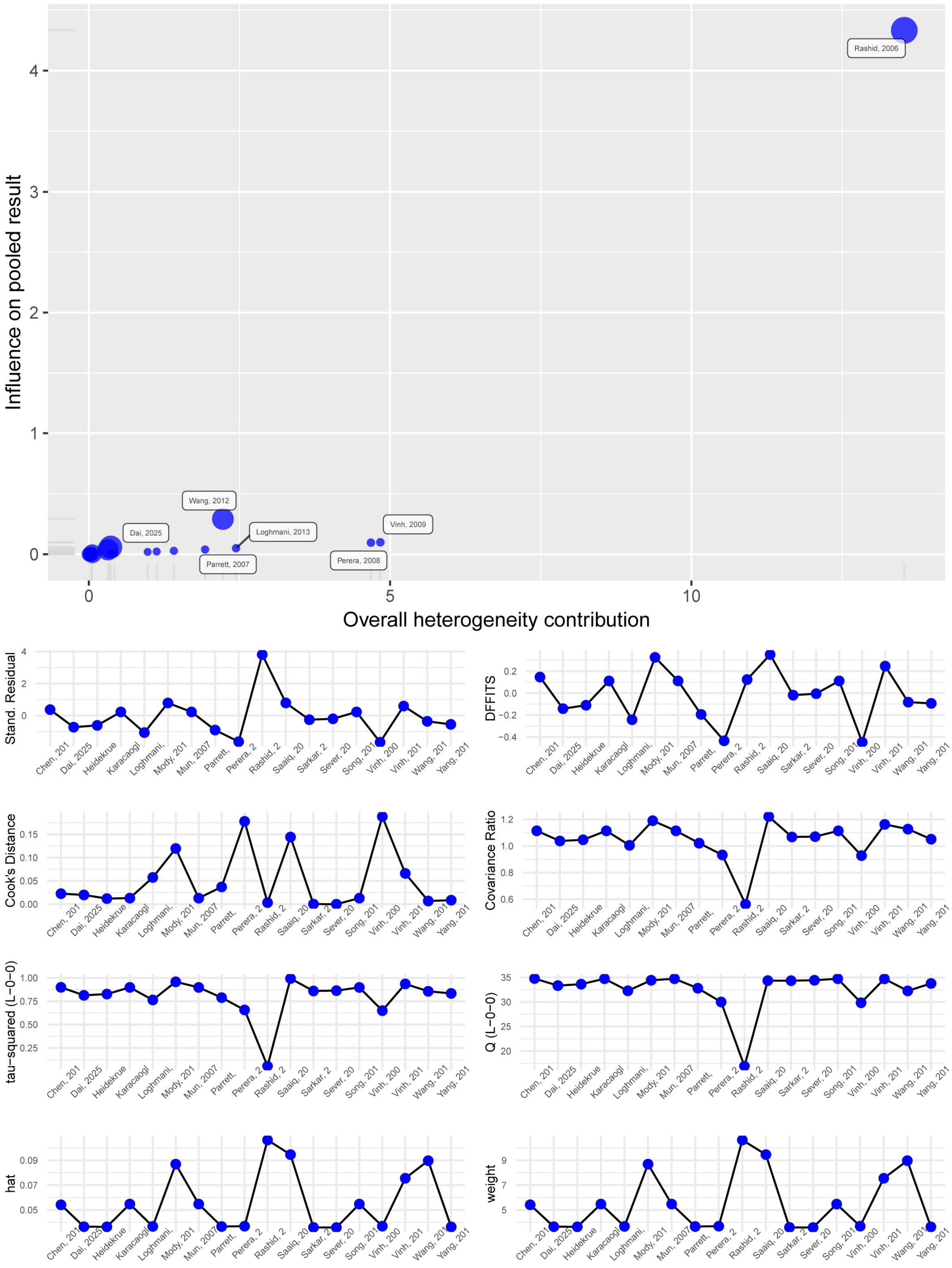

Figure S12. Publication bias for disfiguring scars.

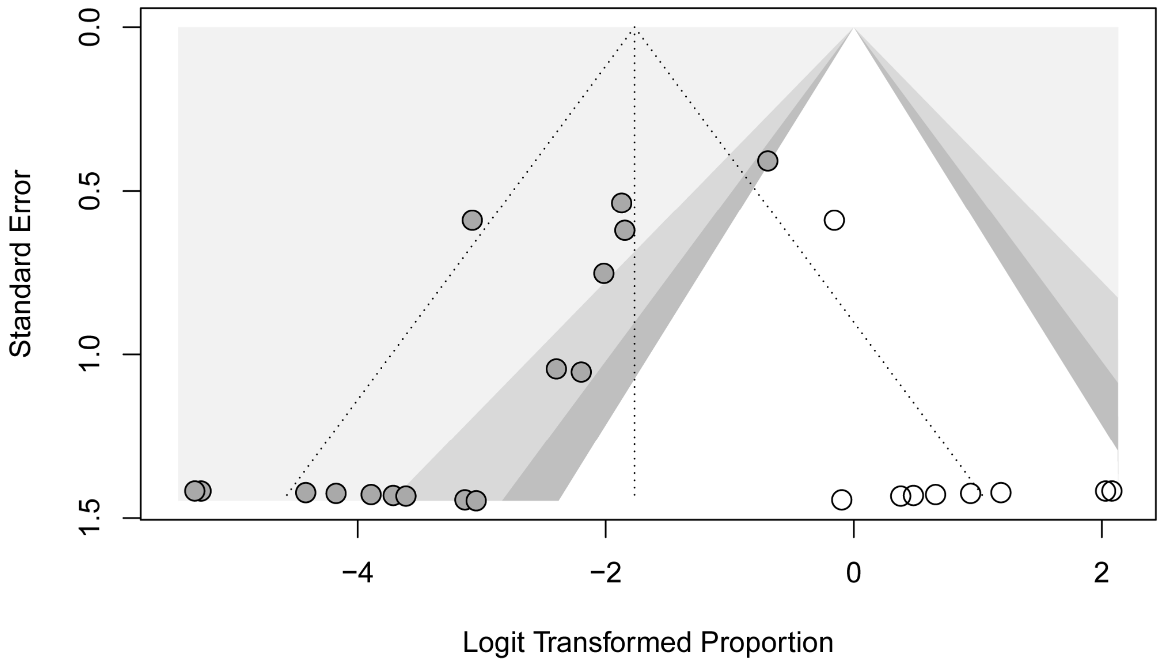

Supplement: Supplementary file 1 [file jcm-15-05583-s001.zip › jcm-4401106-Supplementary- Figures S1-S12-final done.pdf]
