# Supplementary material for: Reconstruction of Post-Burn Neck Contractures: A Systematic Review and Meta-Analysis Comparing Surgical Techniques and Outcomes
Source: J Clin Med. 2026 Jul 16;15(14):5583. doi: 10.3390/jcm15145583 (PMC13413205; doi:10.3390/jcm15145583)
Supplement: Supplementary file 1 [file jcm-15-05583-s001.zip › Supp file. Table S2.pdf]

Supplementary file - Table S2. Influence analysis of studies reporting on contractures occurrence.

**Analysis**	**Proportion (95% CI)**	**95% Prediction Interval**	I^2 **(95% CI)**
Main Analysis	6% (4%; 9%)	0.02; 0.18	26.9% (0; 54.5%)
Influential case removed - Angrigiani, 2017	6% (4%; 9%)	0.02; 0.20	29.7% (0; 56.6%)
