# Supplementary material for: Reconstruction of Post-Burn Neck Contractures: A Systematic Review and Meta-Analysis Comparing Surgical Techniques and Outcomes
Source: J Clin Med. 2026 Jul 16;15(14):5583. doi: 10.3390/jcm15145583 (PMC13413205; doi:10.3390/jcm15145583)
Supplement: Supplementary file 1 [file jcm-15-05583-s001.zip › Supp file. Table S3.pdf]

Table S3. GRADE (Grading of Recommendations Assessment, Development and Evaluation) Summary of Findings

**Outcome**	**No. of studies (participants)**	**Study design**	**Effect (pooled estimate)**	**Heterogeneity (I^2)**	**Certainty of evidence**	**Factors affecting certainty of evidence**
Functional outcome	34 (n=1316)	Observational studies	92% (95%CI 89-95%)	51.0%	Low ⊕⊕◯◯	Observational study design, risk of bias, heterogeneity, publication bias
Aesthetic outcome	20 (n=834)	Observational studies	90% (95%CI 86-94%)	60.9%	Low ⊕⊕◯◯	Observational study design, risk of bias, heterogeneity, publication bias
Complications	45 (n=1599)	Observational studies	15% (95%CI 12-20%)	74.5%	Low ⊕⊕◯◯	Observational design, risk of bias, substantial heterogeneity, publication bias
Recontracture	27 (n=926)	Observational studies	6% (95%CI 4-9%)	26.9%	Low ⊕⊕◯◯	Observational study design, risk of bias, publication bias
Disfiguring scars	18 (n=563)	Observational studies	6% (3-11%)	51.1%	Low ⊕⊕◯◯	Observational study design, risk of bias, heterogeneity, publication bias
